# Case series of coronary artery aneurysms after Everolimus eluting stent implantation and comparison with Sirolimus eluting stents

**DOI:** 10.1186/s12872-022-02503-1

**Published:** 2022-02-17

**Authors:** Raghav Sharma, Aditya Vikram Ruia, Tek Singh Mahant

**Affiliations:** 1 Interventional Cardiologist, HOD Cardiology Unit, Meditrina Hospital (Civil Hospital), Ambala Cantt, Haryana 133001 India; 2 Cardiology Department, Meditrina Hospital (Civil Hospital), 2nd Floor, Ambala Cantt, Haryana 133001 India; 3 Senior Cardiothoracic Surgeon, Executive Director Cardiac Surgery, Fortis Healthcare Hospital, Sector 62, Sahibzada Ajit Singh Nagar, Mohali, Punjab 160062 India

**Keywords:** Coronary artery aneurysm, Xience stent, Methacrylate, Giant aneurysm, Left main aneurysm, Case report, Case series

## Abstract

**Background:**

Coronary artery aneurysms after drug eluting stents are rare. We present a case series of type II coronary aneurysms after implantation of Everolimus eluting stents including patients developing giant aneurysms with a toxic course.

**Case presentation:**

Over a span of 3.5 years at our center 2572 patients were implanted Everolimus eluting stents out of which 4 patients developed coronary type II aneurysms an incidence of 0.00156 whereas 5838 patients were implanted Sirolimus eluting 2nd generation stents out of which 2 patients developed similar aneurysms with an incidence of 0.00034. The slight increase in incidence in Everolimus stents does not reach statistical significance (*p* = 0.054) and is limited by single centre non randomized study. We also propose a hypothesis that the slight increase in the incidence maybe due to allergy to Methacrylate present in Everolimus eluting Xience stent’s primer which is absent in other Sirolimus eluting stents used at our center but that needs to be further investigated. We also found some patients who developed giant aneurysms including Left main aneurysms. In our series operative repair of these patients had better outcomes than covered stent deployment but larger trials maybe needed to confirm the same.

**Conclusions:**

Coronary artery aneurysms after stent implantation are rare but occasionally giant aneurysms are formed with a toxic course. The incidence and morphology of aneurysms after Everolimus and Sirolimus eluting stent deployment do not differ much.

**Supplementary Information:**

The online version contains supplementary material available at 10.1186/s12872-022-02503-1.

## Background

Coronary artery aneurysms are rare, found in 0.3 to 4.9% of patients undergoing coronary angiography. While Kawasaki disease is the most common cause of coronary aneurysm in children, atherosclerosis is the cause in > 90% of adults [1]. The incidence of coronary artery aneurysm after drug eluting stent (DES) deployment varies between 0.2–2.3% and is similar to bare-metal stent (BMS) [2]. Cases have been reported to occur after 3 days to 4 years post percutaneous intervention (PCI). Here we present a case series of coronary aneurysms related to Everolimus eluting second generation stents and compare it with Sirolimus eluting second generation stents at our centre.

## Case presentation

### Initial scheme of treatment at our centre

See Fig. 1.

**Fig. 1:**
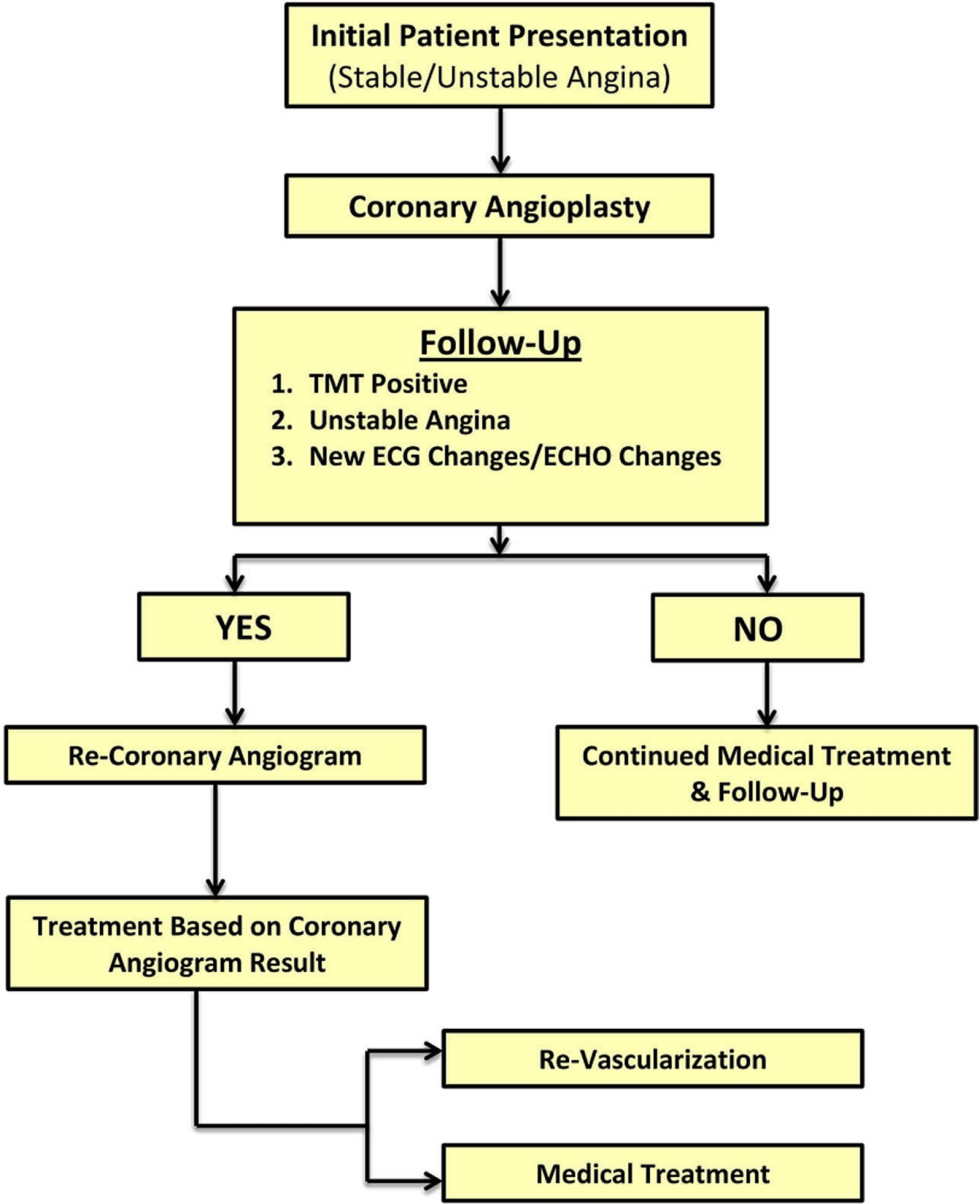
Flowchart of sequence of events at our center

## Case series of aneurysms after Everolimus eluting 2nd generation stents

### Case 1—patient A

**Fig. 2:**
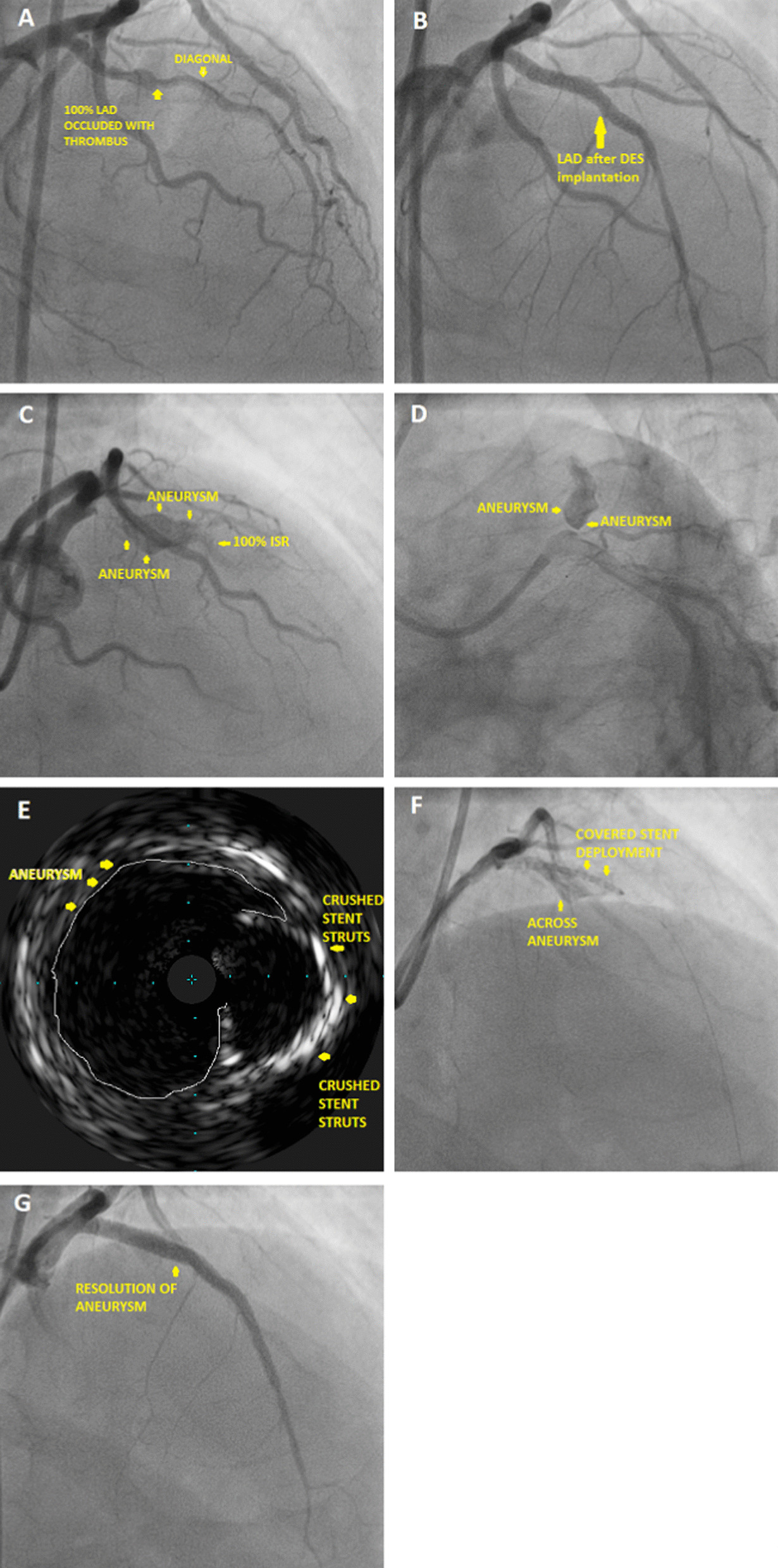
**A** Coronary angiogram showing mid LAD 100% occluded with thrombus, **B** post stenting of LAD with DES, **C** angiogram showing aneurysm in LAD with 100% ISR distally, **D** angiogram showing coronary aneurysm in LAD, **E** intravascular ultrasound showing crushed stent struts with adjoining aneurysm, **F** covered stent deployment across the aneurysm, **G** after covered stent deployment resolution of aneurysm

A 68 year hypertensive male presented with an anterior wall myocardial infarction and was lysed with Streptokinase on Oct 9th, 20. Coronary angiogram (CAG) done (Fig. 2A) showed mid left anterior descending (LAD) artery 100% occluded with thrombus. Subsequently PCI to mid LAD was done using Xience V-2.75 × 28 mm DES (Fig. 2B). The lesion was post dilated with a 3 mm balloon at 15 atm. Subsequently patient presented to us again after 5 months with unstable angina. There was no history of fever during the interim period and total leukocyte count (TLC) on admission was 6600/µL. Echocardiogram (Echo) showed left ventricle ejection fraction (LVEF) of 30%. CAG with intravascular ultrasound (IVUS) was done which showed a large aneurysm in mid LAD (Additional file 1: Video S1, Additional file 2: Video S2, Additional file 3: Video S3) with complete in stent occlusion (Fig. 2C–E). PCI was done with a covered stent 3.5 × 26 mm (Graft master) to ostio-proximal LAD and from proximal to mid LAD with 2.75 × 23 mm Xience V DES (Fig. 2F, G). Subsequently the patient expired after 1.5 months due to diarrhea and sepsis complicating heart failure at an outside hospital.

### Case 2—patient B

**Fig. 3:**
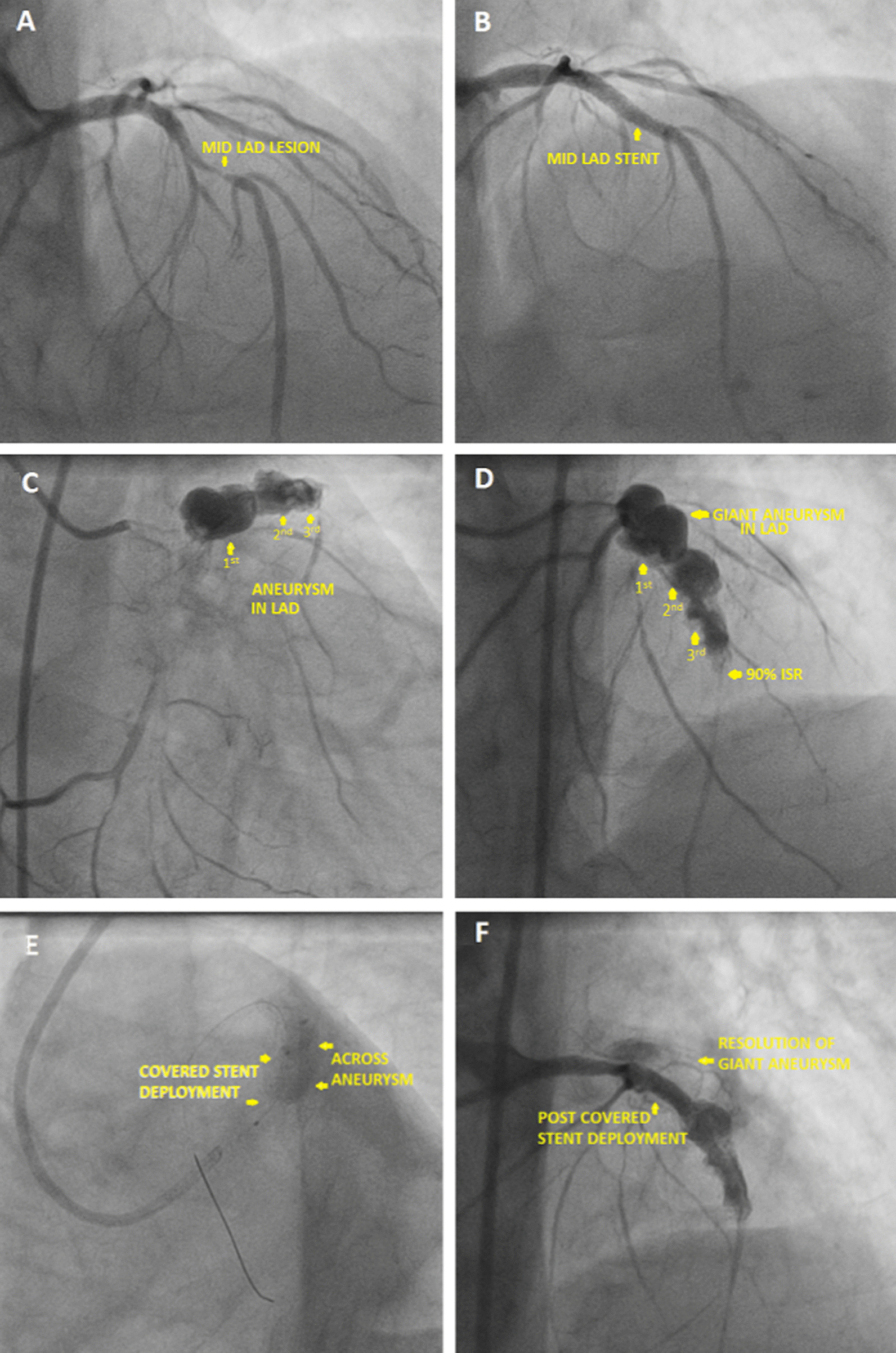
**A** Coronary angiogram showing lesion in mid LAD, **B** post stenting in mid LAD, **C** angiogram showing coronary aneurysm, **D** angiogram showing giant aneurysm in proximal LAD along-with two other saccular aneurysms in LAD, **E** covered stent deployment across the giant aneurysm, **F** post covered stent deployment resolution of giant aneurysm

69 year male with diabetes and hypertension presented with chest pain outside where CAG was done (Fig. 3A) which showed mid LAD 90–95% stenosis, and non-critical lesions in other vessels. Echo showed LVEF 35–40%. Proximal LAD was stented with 2.75 × 33 mm Xience Prime LL deployed at 12 atm (Fig. 3B). Post dilatation was done with 3 × 12 mm NC Traveler balloon at 13 atm. Subsequently the patient presented after 3.5 months with recurrent angina. Echo showed LVEF 35% and TLC was 7900/µL. Repeat CAG showed complete occlusion of LAD stent and three large aneurysms including one giant aneurysm adjacent to the entire length of the stent segment (Fig. 3C, D, Additional file 4: Video S4, Additional file 5: Video S5). So using a covered stent 3.5 × 19 mm (Graft master) the giant aneurysm was approximately safely (Fig. 3E, F) and the patient was scheduled for a staged PCI. Subsequently the patient was lost to follow up and expired after 10 days due to heart failure at an outside hospital.

### Case 3—patient C

**Fig. 4:**
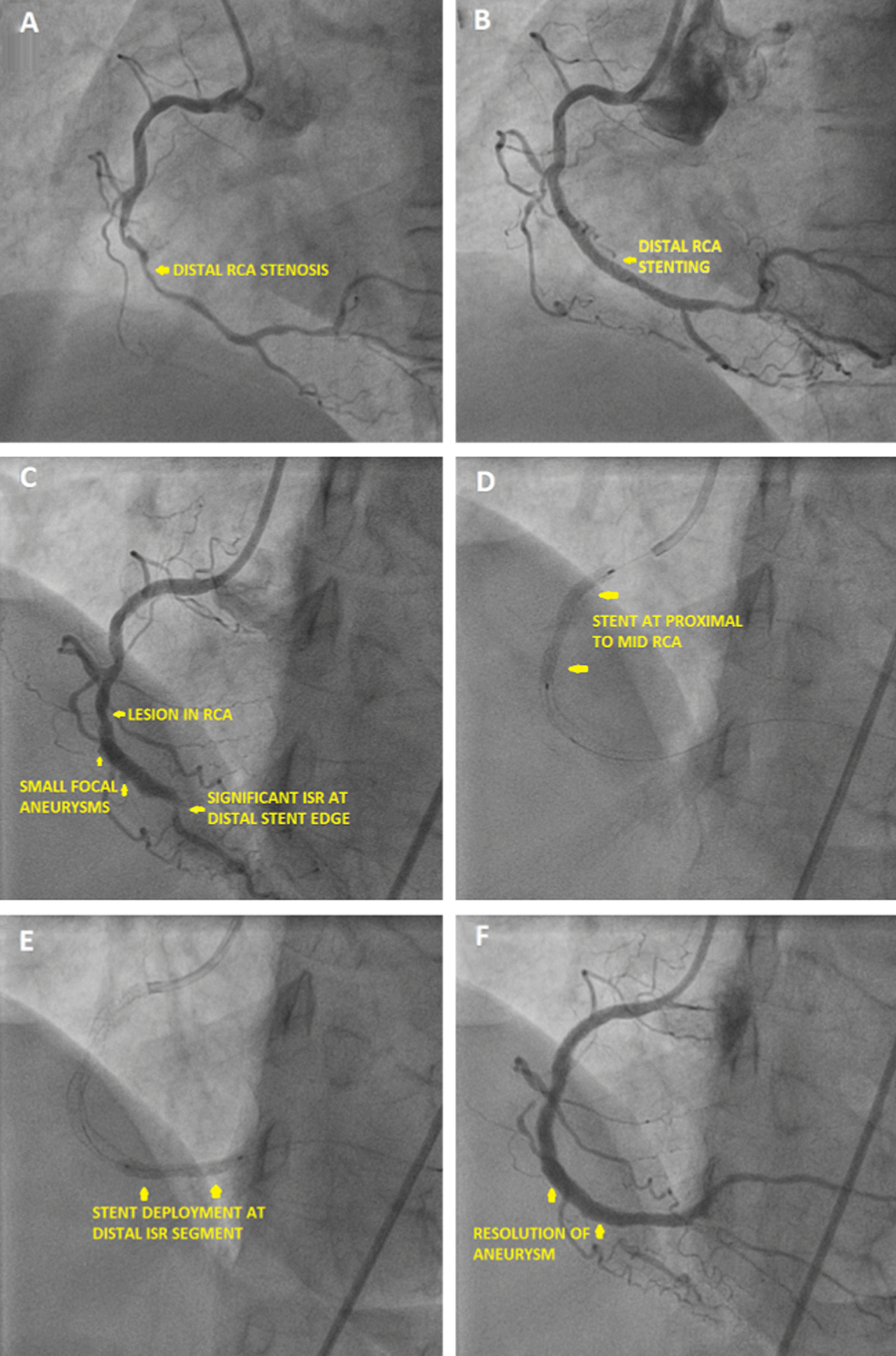
**A** Coronary angiogram showing lesion in distal RCA, **B** coronary angiogram image post stenting in distal RCA, **C** coronary angiogram showing small aneurysms across the stented segment with distal significant ISR and another lesion in proximal RCA, **D** coronary angiogram showing stent deployment in proximal right coronary artery), **E** coronary angiogram showing stent deployment near previous distal ISR, **F** coronary angiogram showing final result with complete resolution of aneurysm

71 year hypertensive female presented to our hospital with unstable angina. CAG (Fig. 4A) showed 70–80% lesion in mid and distal RCA. PCI to mid to distal RCA was done using 2.5 × 38 mm Xience Prime DES (Fig. 4B). The lesion was post dilated using 2.75 × 12 mm NC balloon. Patient was re admitted after 3 months with unstable angina and TLC of 9700/µL. Re-CAG showed proximal RCA 70–80% lesion. The stented segment had multiple small aneurysms seen (Fig. 4C, Additional file 6: Video S6). Distal end of stent had around 98% ISR noted. The RCA proximal lesion was stented with 2.75 × 33 mm Xience Prime DES deployed at 10 atm (Fig. 4D). The distal RCA lesion including the ISR segment was stented with 2.75 × 23 mm Xience V (DES) deployed at 10 atm (Fig. 4E). Post dilatation was done with 3.5 × 12 mm NC Traveler balloon at 15 atm. The final angiogram showed complete resolution of the aneurysms (Fig. 4F). Patient is currently asymptomatic.

### Case 4—patient D

**Fig. 5:**
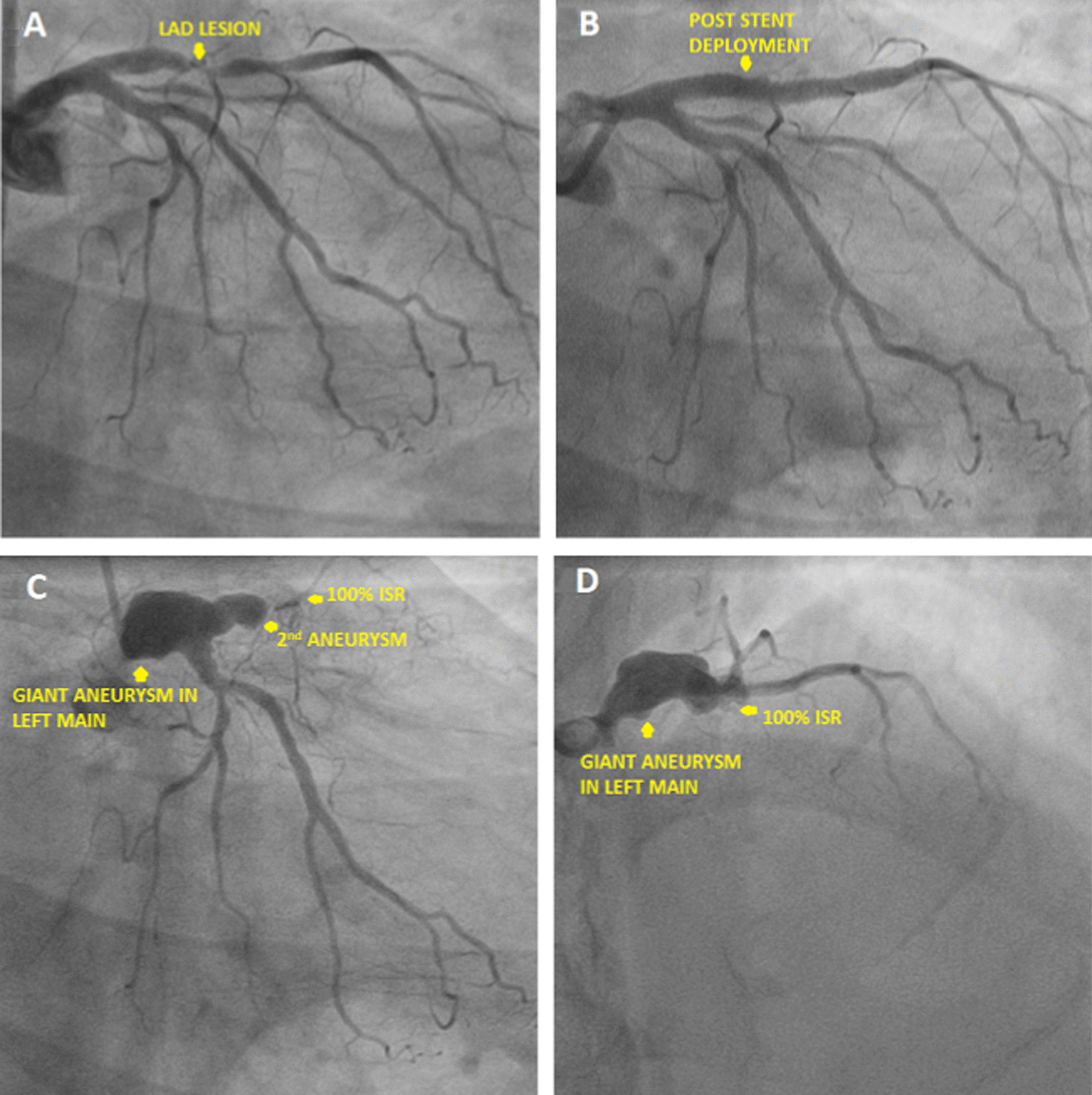
**A** Coronary angiogram image showing lesion in proximal LAD artery, **B** Coronary angiogram image post stenting of proximal LAD, **C** coronary angiogram showing giant aneurysm involving left main artery and 2nd aneurysm in proximal LAD, **D** coronary angiogram showing giant aneurysm in left main artery

54 year diabetic hypertensive male presented to us with unstable angina. CAG done on Oct 9th, 20 showed proximal LAD 99% plaque (Fig. 5A). PCI to LAD was done using 3 × 28 mm Xience V drug eluting stent (Fig. 5B). The lesion was post dilated with 3.5 × 12 mm NC balloon at 15 atm. Patient was re admitted after 8 months with chest pain, LVEF 35% and TLC 8200/µL. CAG repeated showed proximal end of stent in LAD 100% ISR. A giant aneurysm was seen in Left main and one in proximal LAD (Fig. 5C, D, Additional file 7: Video S7). Patient was treated successfully with operative aneurysmal repair and grafting. Intra operatively there was no pus in situ and cultures from the sac were negative. Currently patient is asymptomatic.

## Discussion and conclusions

The mechanism of stent induced aneurysm has been classified by Aoki et al. into 3 types [2]. Type I Aneurysms are formed due to injury to the arterial wall during dissections or high pressure balloon dilatations. These are more often pseudo-aneurysms. IVUS in these cases may demonstrate a contained rupture with thrombus in the aneurysmal sac with only the outer tunica adventitia. These patients present within one month of the index procedure with rapidly enlarging aneurysm and sometimes pericarditis. It is seen more in chronic total occlusions (CTO) and long diffuse lesions where there is inadvertent injury to the sub intimal space.

**Fig. 6:**
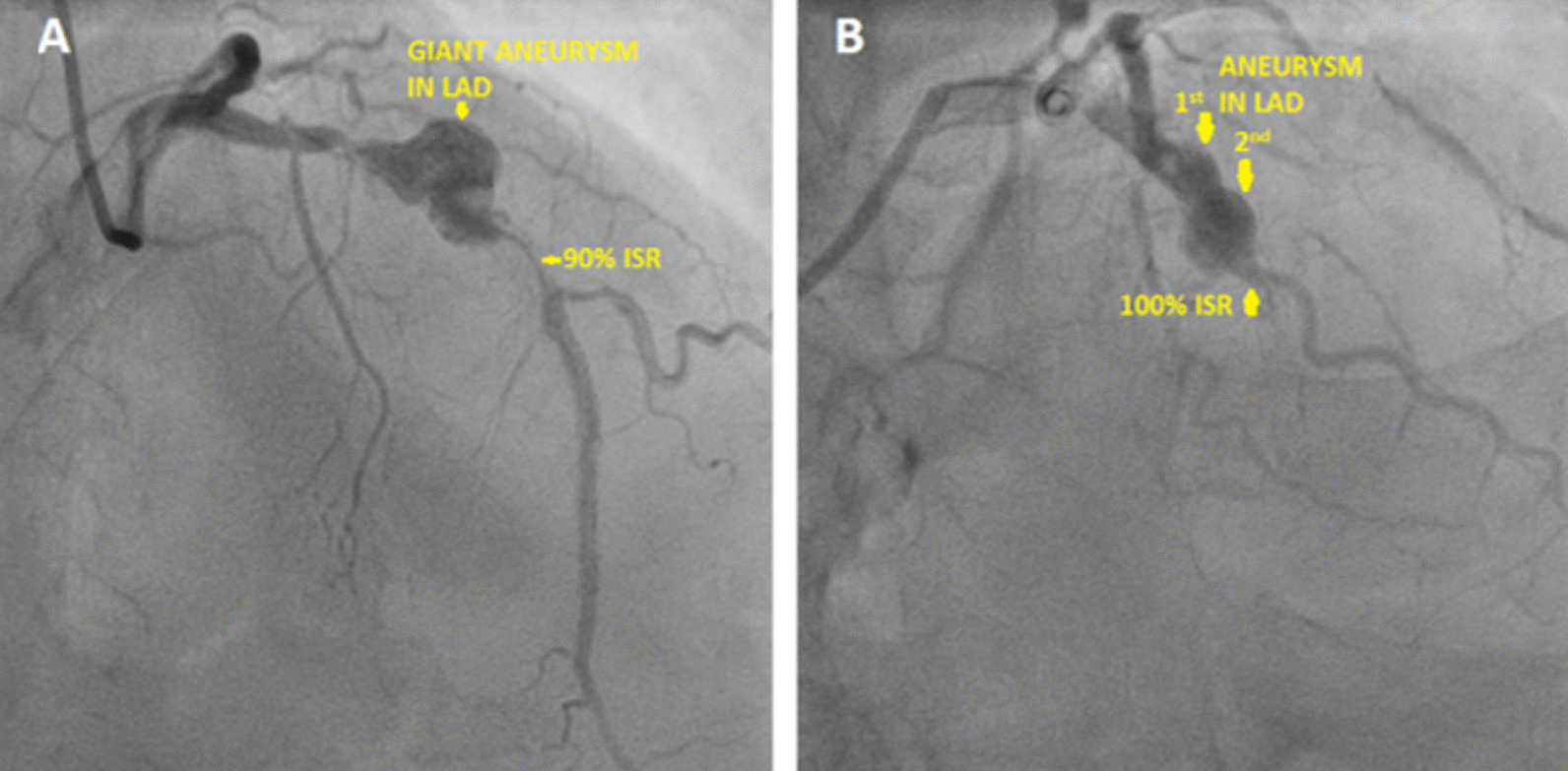
**A** Patient E—patient with Sirolimus eluting 2nd generation stent developing aneurysm, **B** Patient F—patient with Sirolimus eluting 2nd generation stent developing aneurysm

**Fig. 7:**
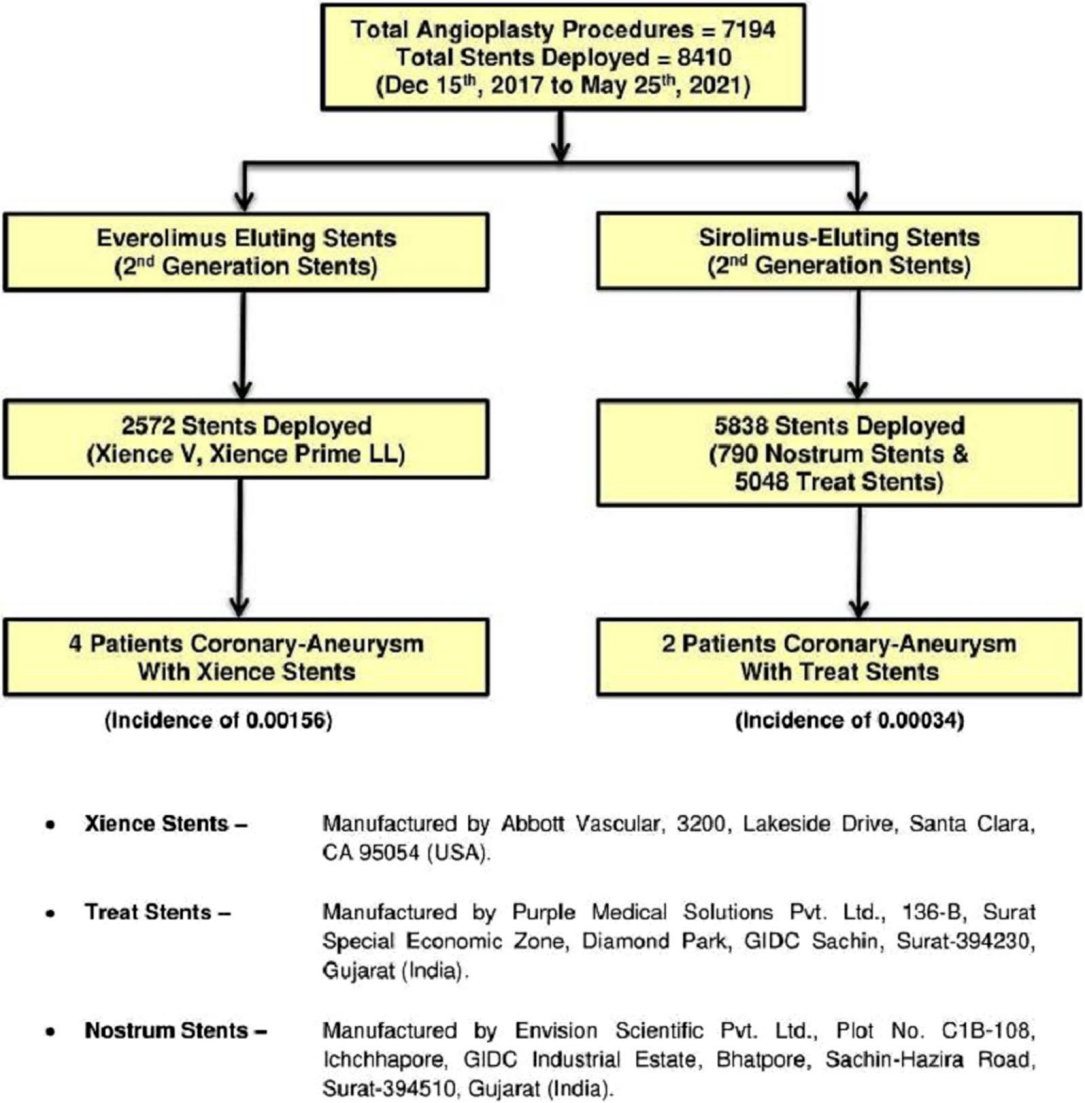
Total angioplasties including various stents used at our center and their incidence of aneurysm formation

Type II stent induced aneurysms present sub acutely due to hypersensitivity reaction to either the stent metal/alloy, the drug polymer or due to the anti-proliferative action of the drug which prevents proper endothelialization. The cobalt chromium stents contain 9–11% nickel compared with stainless steel stents containing 10–14% nickel [3]. Both of them can cause allergy and ISR in patients with comparable rates (2). Here both Xience–Everolimus eluting stents and Treat-Sirolimus eluting stents were used comparably and their baseline characteristics and outcomes have been depicted in Table 1 and Fig. 7. The Xience V/Prime-LL stents used here as has a two layer coating composed of primer layer of PBMA (Poly N Butyl Methacrylate) and a drug reservoir made of poly-vinylidene fluoride-co-hexafluoropropylene (PVDF-HFP) [3]. Fluorinated polymers are bio-compatible as they preferentially adsorb albumin to fibrinogen preventing platelet activation [3]. On the contrary the primer layer containing Methacrylate is known to cause allergic hypersensitivity [5–8]. They have been shown to induce a marked inflammatory reaction consisting primarily of eosinophils and lymphocytes. Sirolimus and Everolimus both prevent neo-intimal proliferation but also delay re endothelialization. This has been a proposing mechanism for abetting aneurysm formation. While both of them are mTORC1 inhibitors, Sirolimus is more protein bound with a longer terminal half-life. Here we also had two patients who developed stent induced aneurysm formation after implantation of 2nd generation Sirolimus eluting stents whose morphology (Fig. 6A, B) were similar to Everolimus eluting 2nd generation stents.

**Table 1:** Properties of Everolimus eluting Xience stents versus Sirolimus eluting Treat stents

	Xience stent (Xience V, Xience Prime LL) [3]	Treat stent [4]
Drug eluted	Everolimus eluting	Sirolimus eluting
Terminal half-life of drug eluted	26–30 h	46–72 h
Generation stent	2nd generation	2nd generation
Stent thickness	81µ	65µ
Stent material	L605 cobalt–chromium alloy	L605 cobalt–chromium alloy
Biocompatibility of stent material	Contains 9–11% nickel, chromium. Can occasionally cause metal allergy	Contains 9–11% nickel, chromium. Can occasionally cause metal allergy
Drug carrier/polymer	Primer layer of PBMA (Poly N Butyl Methacrylate) and a drug reservoir made of poly-vinylidene fluoride-co-hexafluoropropylene (PVDF-HFP)	Biocompatible lactide and glycolide family of biodegradable polymer
Biocompatibility of drug carrier/polymer	**Primer** Methacrylate known to cause hypersensitivity reaction and aneurysm formation **Reservoir** PVDF-HFP is bio compatible	Biocompatible

Figure 7 depicts that the incidence of stent induced aneurysm formation was slightly more with Everolimus eluting Xience stents as compared to Sirolimus eluting 2nd Generation stents (Treat and Nostrum). But by using chi square analysis it does not reach statistical significance (*p* = 0.054).

Type III stent induced coronary aneurysm are infection related mycotic aneurysms. Patients are toxic with bacteremia and have a high blood leukocyte count. In our series none of the patients presented within 1 month of angioplasty with fever or raised TLC and most of them presented with angina. In addition patient D who had operative repair done, no pus was found in the aneurysmal sac. Hence in our series infective etiology was ruled out.

In our case series as depicted in Table 2, most patients were old (average age of 65.5 years), male (75%) and all of them were hypertensive. Possibly hypertension was associated with more arterial shear stress. The native lesions were long segment lesions with near complete occlusion predisposing to intimal injury. LAD was the most common artery involved as it is exposed to higher wall stress during systole and twice the torsion of other arteries. Second generation Everolimus eluting stents were used in this series and the morphology of aneurysms were similar to two patients with Sirolimus eluting 2nd generation stents. There was significant ISR in all of them. In an aneurysm there is slow flow of blood which predisposes to thrombus formation, but IVUS of patient A did not reveal any thrombus. The patients were followed with standard dual anti platelet drugs to minimize stent thrombosis. In patient C the small aneurysms formed were healed after opening the distal ISR segment.

**Table 2:** Baseline characteristics and treatment profile of all patients who developed aneurysms after Everolimus eluting Xience stents

	Patient A	Patient B	Patient C	Patient D
Patient characteristics				
Age	68 years	69 years	71 years	54 years
Sex	Male	Male	Female	Male
Hypertension	Yes	Yes	Yes	Yes
Diabetes mellitus	No	Yes	No	No
Lesion characteristic				
Long/diffuse lesion (> 20 mm)	Yes	Yes	Yes	Yes
% Stenosis	100%	90–95%	80%	99%
Artery involved	LAD	LAD	RCA	LAD
Initial angioplasty details				
Stent size deployed	2.75 × 28 mm	2.75 × 33 mm	2.5 × 38 mm	3 × 28 mm
Stent type	Xience V	Xience Prime LL	Xience Prime LL	Xience V
Metal alloy in stent	L605 cobalt chromium	L605 cobalt chromium	L605 cobalt chromium	L605 cobalt chromium
Drug polymer	Acrylate primer and a fluorinated copolymer	Acrylate primer and a fluorinated copolymer	Acrylate primer and a fluorinated copolymer	Acrylate primer and a fluorinated copolymer
Drug eluting	Everolimus	Everolimus	Everolimus	Everolimus
Post dilatation	3 mm at 15 atm	3 mm at 13 atm	2.75 mm at 16 atm	3.5 mm at 15 atm
Dual anti-platelet drugs	Ticagrelor + ecospirin	Clopidogrel + ecospirin	Clopidogrel + ecospirin	Ticagrelor + ecospirin
Patient re admission presentation				
Fever	Nil	Nil	Nil	Nil
Unstable angina	Yes	Yes	Yes	Yes
Days after initial angioplasty	4 months	3.5 months	2.5 months	7 months
TLC during re-admission	6600/µL	7900/µL	9700/µL	8200/µL
Eosinophil count	600/µL	800/µL	1500/µL	800/µL
Aneurysm details	One large saccular aneurysm	Three large saccular aneurysms	Two–three small fusiform aneurysms	Two large saccular aneurysms
Arterial segment involved	Proximal LAD	Proximal LAD	Distal RCA	Left main and proximal LAD
Second admission treatment				
Angioplasty	Covered stent (3.5 × 26 mm)	Covered stent (3.5 × 19 mm)	Drug eluting stent (2.75 × 23 mm)	Operative repair and grafting
Outcome	Expired	Expired	Alive	Alive

Coronary aneurysm after stent implantation is a grey area and the exact etiology is difficult to define. In our case series mycotic aneurysms are ruled out. There was no instance of dissection or high pressure balloon dilatation moreover we saw more sub-acute diffuse aneurysms including one extending to Left main artery. Therefore type I aneurysms are unlikely. Within type II aneurysm both the stent metal and more commonly the drug polymer causes hypersensitive coronary vasculitis according to various pathological studies. Also patient C had elevated IgE on follow up. While the cobalt chromium alloy can occasionally cause allergy in patients it is commoner in females [9]. In our series patients were predominantly male (75%). Moreover metal allergy predominantly presents as recurrent ISR rather than aneurysm formation [9]. The primer in Xience stents contains Methacrylate (previously used in Cypher stents and also a component of bone cement) which has been known to cause allergic reactions including coronary aneurysm formation in a number of studies [5–8] and we hypothesize that it can be an additional causal agent. Treatment of coronary aneurysms may vary [10]. Small chronic aneurysms can be followed up vigilantly. Whereas giant, enlarging, infected aneurysms presenting acutely should be emergently treated with operative repair [11, 12], covered stent [13–15] or coil. In our study operative aneurysmal repair with grafting had better results especially for giant stent induced type II aneurysms over percutaneous covered stenting.

## Limitations

The major limitations of our study was an absence of histopathological examination of aneurysmal segments to clearly delineate the cause, the use of regionally available stents and limitations related to single centre non randomized study. Also patient A and B had low LVEF and died at an outside hospital so the death was due to heart failure or a possible complication of covered stent deployment cannot be ascertained.

## Conclusion

Our case series highlight two valuable research areas. One is the possibility of methacrylate promoting a hypersensitivity reaction and contributing to formation of Type II stent induced coronary aneurysms. Second is identifying the right patient with giant stent induced type II aneurysm who will benefit from operative repair versus covered stent placement. Both needs to be substantiated by further studies.

## Supplementary Information

**Additional file 1: Video S1.** Angiogram of Patient A showing coronary aneurysm.

**Additional file 2: Video S2.** Angiogram of Patient A showing coronary aneurysm.

**Additional file 3: Video S3.** Intravascular ultrasound of Patient A showing coronary aneurysm.

**Additional file 4: Video S4.** Patient B—Coronary angiogram showing giant aneurysm.

**Additional file 5: Video S5.** Patient B—Coronary angiogram showing giant aneurysm.

**Additional file 6: Video S6.** Patient C—Coronary angiogram showing small aneurysms.

**Additional file 7: Video S7.** Patient D—Coronary angiogram showing giant aneurysm involving left main coronary artery.

## Data Availability

The datasets used and/or analyzed during the current study are available from the corresponding author on reasonable request.

## Abbreviations

IVUS: Intravascular ultrasound
LVEF: Left ventricle ejection fraction
DES: Drug eluting stent
BMS: Bare-metal stent
RCA: Right coronary artery
LAD: Left anterior descending
PCI: Percutaneous coronary intervention
ISR: In stent restenosis
CAG: Coronary angiogram
